# Cytotoxic and genotoxic effects of Br-containing oxaphosphole on *Allium cepa* L. root tip cells and mouse bone marrow cells

**DOI:** 10.1590/S1415-47572009000200028

**Published:** 2009-06-01

**Authors:** Vanya P. Kalcheva, Asya P. Dragoeva, Karamfil N. Kalchev, Dobromir D. Enchev

**Affiliations:** Faculty of Natural Sciences, University of Shumen, ShumenBulgaria

**Keywords:** * Allium cepa L. root tip cells*, Br-containing oxaphosphole derivative, chromosome aberrations, ICR mouse bone marrow

## Abstract

The continuous production and release of chemicals into the environment has led to the need to assess their genotoxicity. Numerous organophosphorus compounds with different structures have been synthesized in recent years, and several oxaphosphole derivatives are known to possess biological activity. Such chemical compounds may influence proliferating cells and cause disturbances of the genetic material. In this study, we examined the cytotoxicity and genotoxicity of 4-bromo-N,N-diethyl-5,5-dimethyl-2,5-dihydro-1,2-oxaphosphol-2-amine 2-oxide (Br-oxph). In *A. cepa* cells, Br-oxph (10^-9^ M, 10 ^-6^ M and 10 ^-3^ M) reduced the mitotic index 48 h after treatment with the two highest concentrations, with no significant effect at earlier intervals. Mitotic cells showed abnormalities 24 h and 48 h after treatment with the two lowest concentrations but there were no consistent changes in interphase cells. Bone marrow cells from mice treated with Br-oxph (2.82 x 10 ^-3^ μg/kg) also showed a reduced mitotic index after 48 h and a greater percentage of cells with aberrations (principally chromatid and isochromatid breaks). These findings indicate the cytotoxicity and genotoxicity of Br-oxph in the two systems studied.

The chemistry of organophosphorus compounds is a subject of increasing interest, and a large number of compounds with different structures, properties and biological activites have been synthesized (Smee and Reist, 1996; Leblond *et al.*, 2002). Heterocyclic organophosphorous compounds are an interesting group of molecules, especially oxaphosphole derivatives that contain oxygen and phosphorus. Enchev *et al.* (1986) demonstrated that some oxaphospholes possess biological activity. Chemical compounds that possess biological activity may influence proliferating cells and cause disturbances of the genetic material. Since many organophosphorus compounds are known to be mutagenic (Lieberman *et al.*, 1998; Blasiak *et al.*, 1999), there is need to screen new organophosphorus compounds for possible genotoxicity.

To assess the potential genotoxicity of any compound, its ability to cause chromosomal damage needs to be evaluated in multiple tests (Repetto *et al.*, 2001). Higher plants provide reliable bioassays for monitoring and testing genotoxins (Grant, 1999), with the *Allium* test being particularly sensitive and reproducible (Fiskesjö, 1985). Small mammals are also useful models for testing genotoxicity (Topashka-Ancheva *et al.*, 2003). The aim of this work was to investigate the cytotoxicity and genotoxicity of 4-bromo-N,N-diethyl-5,5-dimethyl-2,5-dihydro-1,2-oxaphosphol-2-amine 2-oxide (Br-oxph) using *Allium cepa* L. root tip cells and ICR mouse bone marrow cells.

Br-oxph was synthesized in the Laboratory of Organic Chemistry of the University of Shumen (Bulgaria) (Angelov and Enchev, 1987). Since Enchev *et al.* (1986) showed that some oxaphospholes affected plant growth at concentrations of 10^-9^ M, 10^-6^ M and 10^-3^ M, these concentrations were also used in the *Allium* test. The solutions were prepared immediately before use and the cells were incubated with Br-oxph for 3 h and then for a further 24 h and 48 h in the absence of the compound. The 3 h incubation was used since this period corresponded to the earliest appearance of DNA damage (Williams and Omoh, 1996; Miyamae *et al.*, 1997).

Thirty *Allium cepa* L. cv. Stuttgarter Riesen seeds (2n = 16) purchased from a local market were placed on filter paper in Petri dishes containing 5 mL of distilled water and the dishes were then sealed and incubated at 25 ± 1 °C for 72 h. Germinated seeds with roots of equal length (~1 cm) were used in three experiments. In the first experiment, 5 mL of Br-oxph (10^-9^ M, 10^-6^ M or 10^-3^ M) was added to the dishes followed by incubation for 3 h at 25 ± 1 °C. In the other two experiments, after the 3 h incubation described above, the seedlings were removed and placed on filter paper in Petri dishes containing 5 mL of distilled water and incubated for a further 24 h or 48 h at 25 ± 1 °C in the absence of Br-oxph to assess their ability to recover from possible damage. Distilled water and methyl methanesulfonate (MMS, CAS 66-27-3; 10^-4^ M for 24 h) were used as negative and positive controls, respectively.

Chromosomal aberrations in *Allium* root cells were assessed by light microscopy (Rank, 2003). The roots were fixed in Clarke's fixative (95% ethanol:acetic acid glacial, 3:1 v/v) for 90 min, hydrolyzed in 3 N HCl for 8 min and in 45% acetic acid for 30 min at room temperature, and stained for 30 min in 1% aceto-orcein. The terminal root tips (1-2 mm) were removed and squashed in 45% acetic acid. The microscopic analysis included calculation of the mitotic index and the scoring of aberrant cells. Each sample consisted of six root meristems with at least 600 cells analyzed in each meristem. The mitotic index was calculated by counting the number of mitotic cells in 100 cells/root. The categories of aberrations scored included chromosomal bridges and fragments, vagrant chromosomes, aberrant metaphases and anaphases in dividing cells, micronuclei in interphase cells, and the presence of binucleate cells.

ICR mice (2n = 40) were obtained from the Base for Experimental Animals at Slivnitza (Bulgaria). All of the experiments were done under permission granted by the Faculty of Natural Sciences of the University of Shumen (Bulgaria) (permission no. 153/02). The mice were housed on a 12/12 h light/dark cycle at 24° ± 2 °C, with access to water and food *ad libitum*. Eleven experimental groups, each containing 10 mice (5 males and 5 females) were used. The mice were injected with Br-oxph (1 mL per 100 g/bw, i.p., of 10^-3^ M, 10^-6^ M and 10^-9^ M solutions that corresponded to doses of 2.82 x 10^3^ μg/kg, 2.82 μg/kg and 2.82 x 10^-3^ μg/kg, respectively). Saline (NaCl, 0.9% w/v) and MMS (1.10 x 10^2^ μg/kg bw) were used as negative and positive controls, respectively. The mice were killed 3 h, 24 h and 48 h after the administration of Br-oxph, and the controls were killed 24 h after the administration of 0.9% NaCl or MMS.

Bone marrow cell preparations were prepared essentially as described by Preston *et al.* (1987). The mice were injected with colchicine (4 mg/kg, i.p.) and 90 min later they were killed by cervical dislocation. The femurs were removed and the cells were flushed out with 0.075 M KCl and incubated in the same hypotonic solution for 25 min at 37 °C. The lysed cells were then fixed in methanol:acetic acid (3:1, v/v), air dried and stained with 5% Giemsa stain. The mitotic index was calculated by counting the number of mitotic cells in 1000 cells per mouse. Fifty well-spread metaphases per mouse were analyzed for chromosomal aberrations (Preston *et al.*, 1987), using the following categories: chromatid and isochromatid breaks, centromeric and telomeric fusions, and fragments. The number of tetraploid metaphases (as a result of spindle abnormalities), chromosome gaps (defined as achromatic lesions; Ito and Ito, 2001) and apoptotic cells (identified by typical fragmented condensed nuclei; Gorneva *et al.*, 2005) were also determined.

The results were expressed as the mean ± standard deviation (SD) and statistical comparisons were done by using Student's *t*-test, with p < 0.05 indicating significance.

Table 1 shows that there was no significant change in the mitotic index of *Allium* root cells immediately after (0 h) and 24 h after a 3 h incubation with Br-oxph. In contrast, there was a significant reduction in the mitotic index 48 h after a 3 h incubation with the two highest concentrations (10^-6^ M and 10^-3^ M) of Br-oxph. Br-oxph induced a variety of chromosomal aberrations in mitotic cells of *A. cepa* L. root tips (Figure 1) in which anaphases with spindle abnormalities and anaphases and telophases with vagrant chromosomes were the most frequent alterations; anaphase/telophase fragments and bridges and C-mitoses were less frequent.

The treatment with 10^-9^ M Br-oxph significantly increased the percentage of chromosomal aberrations in mitotic cells (by ~7-fold) compared to the controls (Table 1), but there were no significant changes with the two higher concentrations. During the 24 h recovery period after the 3 h incubation with 10^-9^ M and 10^-6^ M Br-oxph, the percentage of chromosomal aberrations was ~5-fold higher than in the control cells; the highest concentration (10^-3^ M) did not significantly affect the number of these aberrations. Similar responses were seen after the 48 h recovery period, although the increases were greater. Abnormalities (the presence of micronuclei and binucleate cells) were also seen in interphase cells (Figure 1). There were no significant changes in the percentage of abnormal interphase cells immediately after the 3 h incubation or during the 24 h and 48 recovery periods, except for an increase with 10^-3^ M Br-oxph after 24 h.

Br-oxph triggered apoptosis in bone marrow cells 3 h after administration. Light microscopy revealed typical signs of apoptosis, *i.e.*, nuclear fragmentation and condensation (Figure 2), and this apoptotic effect persisted up to 48 h after treatment. Chromosomal aberrations were scored only when the frequency of mitotic cells was enough for determination of at least 50 well spread metaphases per animal. When apoptosis was very extensive, it was impossible to determine the mitotic index and chromosomal aberrations. For this reason, Table 2 shows only the effect of the lowest dose of Br-oxph tested (2.82 x 10^-3^ μg/kg; 24 h and 48 h after the treatment). This dose significantly reduced the mitotic index by 55% 48 h after the treatment, and there was a significant increase (2-fold) in the level of chromosomal aberrations in mitotic cells 24 h and 48 h after the injection of Br-oxph (Table 2). Chromatid breaks were the most frequent aberrations, but isochromatid breaks and centromeric and telomeric fusions were also observed; fragmentation was seen only 48 h after treatment. There was no change in the percentage of cells with gaps or in the number of tetraploid cells.

These results show that in both systems Br-oxph depressed cellular proliferation (mitosis) 24 h and 48 h after treatment. Interestingly, Br-oxph (10^-6^ M and 10^-3^ M) appeared to stimulate cell division in *A. cepa* root tips immediately after a 3 h treatment, but this effect was transitory and was not seen after 24 h and 48 h. The decrease in the mitotic index indicates that Br-oxph can arrest cell growth. The suppression of mitotic activity is often used to assess cytotoxicity (Smaka-Kincl *et al.*, 1996). The ability of Br-oxph to induce chromosomal aberrations in *A. cepa* root tips and bone marrow cells after treatment for 3 h and during 24 h and 48 h of recovery agrees with the findings of Williams and Omoh (1996) and Miyamae *et al.* (1997), who observed DNA damage after a 3 h exposure to other compounds.

Br-oxph was generally less genotoxic than the positive control (MMS). The abnormalities caused by Br-oxph showed little concentration- or time-dependence. Interestingly, 10^-3^ M Br-oxph caused fewer aberrations in *A. cepa* immediately after the treatment and during the 24 h recovery than did concentrations of 10^-9^ M and 10^-6^ M. A lack of concentration-dependence in the effects of other oxaphospholes has also been reported (Enchev *et al.*, 1986). In the case of Br-oxph, the nonlinear relationship may reflect the influence of this compound on cell division. The increase in the percentage of chromosomal aberrations in *A. cepa* mitotic cells 48 h after treatment with 10^-6^ M and 10^-3^ M Br-oxph correlated with the inhibition of cell division. A number of factors, such as compound solubility, rate of transport and biodistribution, and concentration at the target site (which is influenced by time and cellular permeability), can modulate the time of occurrence of chemically-induced aberrations (McFee and Tice, 1990). In addition, there was marked individual variation in the responses to Br-oxph, which meant that in some experiments the changes observed were not significant.

There were differences in the chromosomal aberrations caused by Br-oxph in the two test systems. The occurrence of abnormal anaphases and C-mitosis in *A. cepa* indicated that spindle formation was adversely affected (El-Ghamery *et al.*, 2000). According to Rank (2003), vagrant chromosomes are also indicators of spindle poisoning. In bone marrow cells, chromatid breaks were the most frequent aberrations, whereas the number of tetraploid metaphases in bone marrow cells was unaffected by the treatment. These findings suggest a plant-specific action of Br-oxph on spindle formation.

Fusion between chromatids can be initiated by the simultaneous breakage of two chromatids or by the loss of telomere capping (Gilley *et al.*, 2005). The bridges seen in *A. cepa* cells were also probably formed by breakage and fusion of chromosomes and chromatids (Türkoglu, 2007). The relatively low percentage of bridges and fragments in *A. cepa* root tips agreed with the relatively low percentage of cells with micronuclei (Krishna and Hayashi, 2000). The detection of a binucleate condition in *Allium* indicated that Br-oxph solutions inhibited cytokinesis.

Apoptosis is an energy-dependent, genetically controlled process by which unnecessary or damaged cells die (Martin, 1993; Earnshaw, 1995). DNA damage can induce cell death and thus, the occurrence of apoptosis in mouse bone marrow cells was another indication of the cytotoxicity of Br-oxph. Light microscopy showed the presence of apoptotic nuclei with an altered morphology with typical nuclear fragmentation and condensation, as described by others (Kam and Ferch, 2000; Gorneva *et al.*, 2005). According to Grishin *et al.* (2001), genotoxic stresses activate intracellular signaling molecules, which lead to growth arrest, DNA repair, and/or apoptosis. Although several of the pathways linking DNA damage to mitochondria-dependent and -independent mechanisms of death have been elucidated, the connectivity of these pathways is subject to regulation by various other poorly understood factors (Borges *et al.*, 2008).

In conclusion, the results of this study indicate cytooxicity and genotoxicity of Br-oxph in *A. cepa* root tip cells and ICR mouse bone marrow cells, with the effects being observed up to 48 h after treatment for 3 h. Chromosomal aberrations provide a sensitive endpoint for assessing the genotoxicity of chemicals (Topashka-Ancheva *et al.*, 2003) and, as shown here, *A. cepa* may be a sensitive biosensor for screening the genotoxicity of oxaphospholes. On the other hand, our data are in accordance with observation that rodent bioassays are useful for investigating the pharmacokinetics, mechanisms of action, and differential toxicity of various chemicals (Roldan-Arjona *et al.*, 1991).

**Figure 1:**
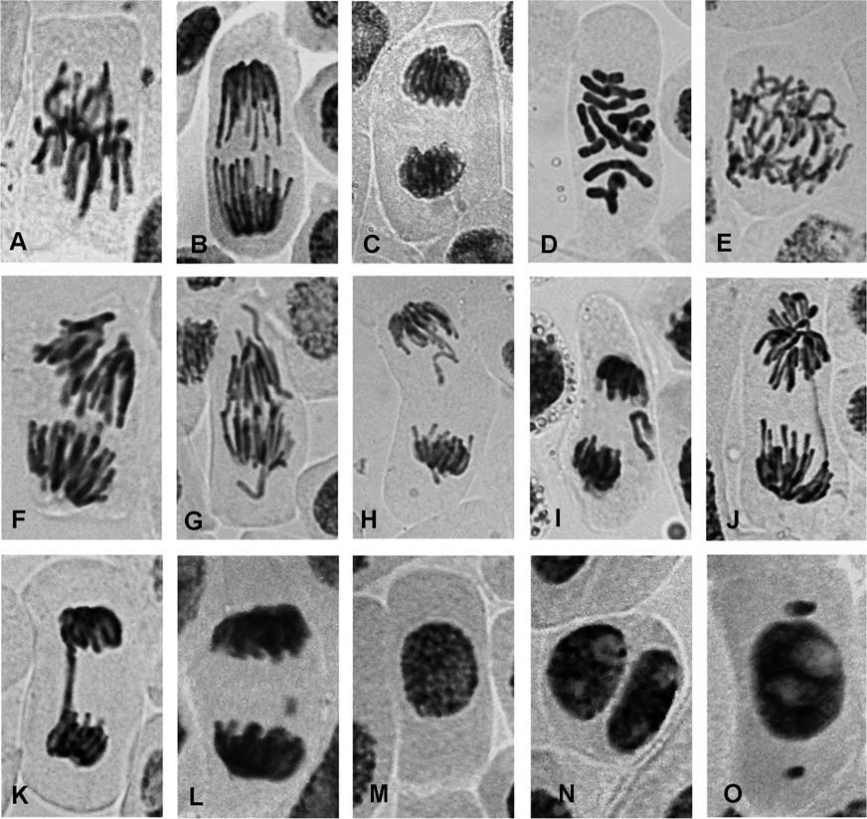
Aberrations induced by Br-oxph in *Allium cepa* root tips: A - normal metaphase, B - normal anaphase, C - normal telophase, D,C - mitosis, E, F - Spindle abnormalities in anaphase, G - vagrant chromosome in anaphase, H, I - vagrant chromosome in anaphase-telophase, J - anaphase bridge, K - anaphase-telophase bridge, L - anaphase-telophase with fragment, M - normal interphase cell, N - binucleated cell, O - micronuclei in interphase cell.

**Figure 2:**
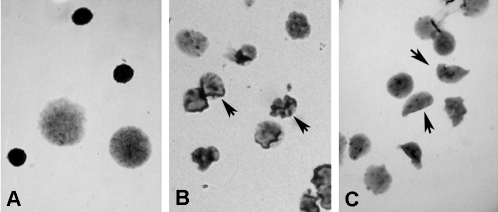
Bone marrow nuclei of ICR mice treated with Br-oxph showed a typical apoptotic morphology that included fragmentation and condensation: A - normal nucleus, B - condensed nucleus, and C - fragmented nucleus.

## Figures and Tables

**Table 1:** Mitotic index and abnormalities in mitotic and interphase cells in root tip meristems of *Allium cepa* L. incubated with Br-oxph (10^-9^, 10^-6^ or 10^-3^ M) for 3 h followed by no recovery interval (0 h) or by recovery for 24 h and 48 h.

Recovery time (h)	Sample	Mitotic index (%)	Abnormalities in mitotic cells (% total)	Abnormalities in interphase cells (% total)
0	NC	6.79 ± 2.31	1.94 ± 1.13	0.16 ± 0.26
	10^-9^ M	6.54 ± 1.31	12.64 ± 8.06**	0.50 ± 0.32
	10^-6^ M	7.72 ± 2.00	4.71 ± 3.64	0.62 ± 0.55
	10^-3^ M	7.56 ± 2.35	4.24 ± 3.02	0.11 ± 0.14

24	NC	7.83 ± 2.60	2.01 ± 2.61	0.09 ± 0.09
	10^-9^ M	6.10 ± 1.29	9.45 ± 5.66*	0.25 ± 0.39
	10^-6^ M	6.24 ± 2.18	10.34 ± 4.25**	0.43 ± 0.63
	10^-3^ M	5.63 ± 2.67	4.25 ± 4.37	0.54 ± 0.48*
	PC	3.86 ± 1.49**	20.30 ± 11.27**	1.11 ± 0.73**

48	NC	6.61 ± 1.08	1.46 ± 1.74	0.22 ± 0.29
	10^-9^ M	6.03 ± 0.99	12.39 ± 0.55***	0.45 ± 0.54
	10^-6^ M	4.14 ± 2.41*	13.24 ± 4.23***	0.19 ± 0.48
	10^-3^ M	3.51 ± 1.64**	11.11 ± 12.87	0.06 ± 0.09

The results are expressed as the mean ± SD. *p ≤ 0.05, **p ≤ 0.01 and ***p ≤ 0.001 compared to the corresponding negative control (NC; distilled water). PC - positive control (methyl methanesulfonate, 10^-4^ M).

**Table 2:** Cytogenetic analysis of mouse bone marrow cells 24 h and 48 h after treatment with Br-oxph (2.82 x 10^-3^ μg/kg).

Dose (μg/kg)	Time after treatment (h)	Mitotic index (%)	Metaphases scored	Type of aberration					Cells with aberrations (%)^#^	Cells with gaps (%)	Tetraploid metaphases (%)
				ICB	CB	c/c	t/t	Fr			
NC	24	1.97 ± 1.03	500	1	21	1	1	0	4.40 ± 3.10	3.00 ± 2.71	1.20 ± 1.69
2.82 x 10^-3^	24	1.48 ± 1.13	350	5	28	2	1	0	9.14 ± 3.44*	1.71 ± 2.14	0.00 ± 0.00*
	48	0.89 ± 0.57*	350	4	31	1	0	4	10.80 ± 6.57*	2.00 ± 1.15	0.29 ± 0.76
PC	24	0.67 ± 0.29**	400	28	106	1	2	0	20.44 ± 8.99***	2.25 ± 1.98	0.44 ± 1.33

The results are expressed as the mean ± SD. *p ≤ 0.05, **p ≤ 0.01, and ***p ≤ 0.001 compared with the corresponding negative control (NC; 0.9% NaCl). ^#^Some metaphases had more than one aberration. CB - chromatid breaks, c/c - centromeric fusions, Fr - fragments, ICB - isochromatid breaks, t/t - telomeric fusion, PC - positive control (methyl methanesulfonate, 1.10 x 10^2^ μg/kg bw).
